# Unmasking nonspecific pain – clinical clues and diagnostic challenges in acute aortic syndromes: Case series

**DOI:** 10.1097/MD.0000000000046200

**Published:** 2025-11-21

**Authors:** Ladislav Kočan, Dušan Rybár, Adrián Kolesár, Štefan Lukačin, Alena Šujanová, Andrea Galovičová, Jozef Židzik, Janka Vašková

**Affiliations:** a Department of Anaestesiology and Intensive Medicine, Faculty of Medicine, Pavol Jozef Šafárik University in Košice, Košice, Slovak Republic; b Department of Critical Care, East Slovak Institute for Cardiovascular Diseases, Košice, Slovak Republic; c Department of Cardiac Surgery, East Slovak Institute for Cardiovascular Diseases, Košice, Slovak Republic; d Department of Heart Surgery, Faculty of Medicine, Pavol Jozef Šafárik University in Košice, Košice, Slovak Republic; e ProCare, Pain Clinic in Košice, Košice, Slovak Republic; f Department of Imaging Techniques, East Slovak Institute for Cardiovascular Diseases, Košice, Slovak Republic; g Department of Medical Biology, Faculty of Medicine, Pavol Jozef Šafárik University in Košice, Košice, Slovak Republic.

**Keywords:** aneurysm, aortic dissection, aortic pathology, vertebrogenic pain

## Abstract

**Rationale:**

Acute aortic syndromes, including dissection and aneurysm, are life-threatening vascular emergencies that often present with vague, nonspecific pain. Such pain may mimic common musculoskeletal or visceral disorders such as vertebrogenic, renal, or gastrointestinal pain, leading to diagnostic delays and adverse outcomes. This case series underscores how atypical vertebrogenic-like pain can conceal underlying aortic pathology and highlights the importance of early imaging in suspicious cases.

**Patient concerns:**

A 56-year-old man with descending thoracoabdominal aortic aneurysm, and 35-year-old man with Stanford Type A ascending aortic dissection.

**Diagnoses:**

CT angiography of the aorta due to persistent vertebrogenic back pain despite treatment of lumbar spine pathology revealing a large descending thoracoabdominal aortic aneurysm. In second case a CT scan of the cervical and thoracic spine performed after persistent pain despite cervical vertebrogenic syndrome analgesics treatment led to aortic dissection confirmation.

**Interventions:**

Elective surgical repair of aneurysm via thoraco-phreno-laparotomy. The aneurysm was resected and replaced with a 22-mm aorto-aortic Dacron graft. In second case, an emergency surgery with veno-arterial extracorporeal membrane oxygenation support with an open chest due to ongoing uncontrollable hemorrhage was provided.

**Outcomes:**

Patient was discharged on postoperative day 7 in stable condition. A second patient passed away of early complications as systemic inflammatory response syndrome, acute renal failure, and circulatory shock.

**Lessons:**

These cases emphasize the diagnostic challenges of aortic pathologies in the context of misleading clinical features. Physicians must maintain a high index of suspicion for acute aortic syndromes, particularly when encountering atypical or refractory pain in patients with cardiovascular risk factors. Early diagnosis and adherence to guideline-based management are critical to improving survival in these high-risk emergencies.

## 1. Introduction

Pain is frequently the first symptom of aortic dissection or aneurysmal rupture; however, its nonspecific nature often obscures the underlying vascular emergency. In particular, thoracic or abdominal pain may closely resemble vertebrogenic syndromes, leading to misinterpretation as spinal or musculoskeletal pathology. This overlap highlights the need for heightened diagnostic vigilance, especially in patients with cardiovascular risk factors and atypical or disproportionate pain presentations. Aortic dissection and aneurysm rank among the most critical aortic diseases, both arising from structural weakening of the arterial wall. Dissection involves an intimal tear with subsequent formation of a false lumen between the tunica intima and tunica media, while an aneurysm represents a pathological, persistent dilation of the vessel that may eventually rupture.^[1,2]^

Aortic dissection is classified under acute aortic syndromes (AAS) and is categorized clinically according to the Stanford classification to Type A - involves the ascending aorta and requires emergent surgical repair, and Type B which is confined to the descending aorta distal to the left subclavian artery and is typically managed conservatively or via endovascular intervention.^[3,4]^ Dissections can extend into branch vessels supplying the heart, brain, kidneys, or intestines, resulting in ischemia and organ dysfunction. The most common underlying etiologies include chronic hypertension, cystic medial necrosis, connective tissue disorders (e.g., Marfan syndrome), and iatrogenic trauma during catheter-based procedures.^[5]^

On the contrary, abdominal aortic aneurysms (AAA) typically evolve as chronic conditions, primarily due to atherosclerosis. AAA are most often chronic, degenerative lesions of the aortic wall, usually related to atherosclerosis, and typically enlarge gradually over many years. Nevertheless, they may occasionally present acutely, for example in the setting of abrupt blood pressure elevation or impending rupture. According to current international guidelines, AAA are defined as a permanent dilation of the abdominal aorta measuring ≥ 30 mm in diameter, corresponding to approximately 1.5 times the normal infrarenal aortic diameter.^[6,7]^ The risk of rupture increases sharply once the aneurysm surpasses 55 mm, with an annual risk of over 10%.^[8]^ Aneurysms may remain asymptomatic until rupture. When symptomatic, typical signs include abdominal pain radiating to the back, a pulsatile abdominal mass, and hemodynamic instability. Rupture leads to massive hemorrhage into the retroperitoneal or peritoneal cavity, rapidly progressing to hypovolemic shock.^[9]^ Histomorphologically, aneurysms are classified as true aneurysms, involving all 3 layers of the arterial wall; dissecting aneurysms, associated with intimal tearing and false lumen formation; and false (pseudo)aneurysms, resulting from full-thickness arterial wall disruption contained by perivascular connective tissue. Management of aortic pathology includes open surgery or endovascular repair, with TEVAR used for thoracic aneurysms and EVAR for abdominal aneurysms, depending on anatomy and urgency.^[10,11]^

Both aortic dissections and aneurysms are life-threatening conditions with high mortality if not promptly diagnosed and managed. A major clinical challenge lies in their often misleading symptomatology, which may mimic more benign conditions such as acute coronary syndrome, renal colic, diverticulitis, or vertebrogenic pain. Misinterpretation or underestimation of these symptoms may delay diagnosis and allow disease progression, with catastrophic consequences. Effective management requires timely recognition, adherence to structured diagnostic algorithms, and close multidisciplinary collaboration.^[12]^ The following case reports illustrate clinical scenarios in which accurate pain assessment led to life-saving treatment – or, tragically, proved insufficiently early in critical situations.

## 2. Case presentations

### 2.1. *Case report 1* – *descending thoracoabdominal aortic aneurysm with atypical presentation*

A 56-year-old man was referred to a pain clinic following orthopedic and urologic evaluations for lower back pain persisting for approximately 2 weeks. His medical history included arterial hypertension, hyperlipidemia, nephrolithiasis, and smoking. The pain was described as dull and pressure-like, without radicular features, and worsened with coughing and sneezing. Lumbar spine X-ray revealed osteochondrosis at the L2/3 and L5/S1 levels, without evidence of listhesis or vertebral compression. The patient underwent several follow-up orthopedic evaluations and was repeatedly diagnosed with lumbosacral syndrome (LIS) without root involvement. Treatment included analgesics (tramadol, metamizole, meloxicam) and topical nonsteroidal anti-inflammatory ointments. Despite therapy, a pain management assessment revealed persistent pain rated at 3 to 4/10 on the numerical rating scale, along with nocturnal awakenings and disrupted sleep. Due to his urological history and ultrasound findings of left-sided hydronephrosis, he was also evaluated by a urologist, who noted pelvic dilatation and a hyperechoic lesion suspicious for a renal calculus.

**Figure 1.**
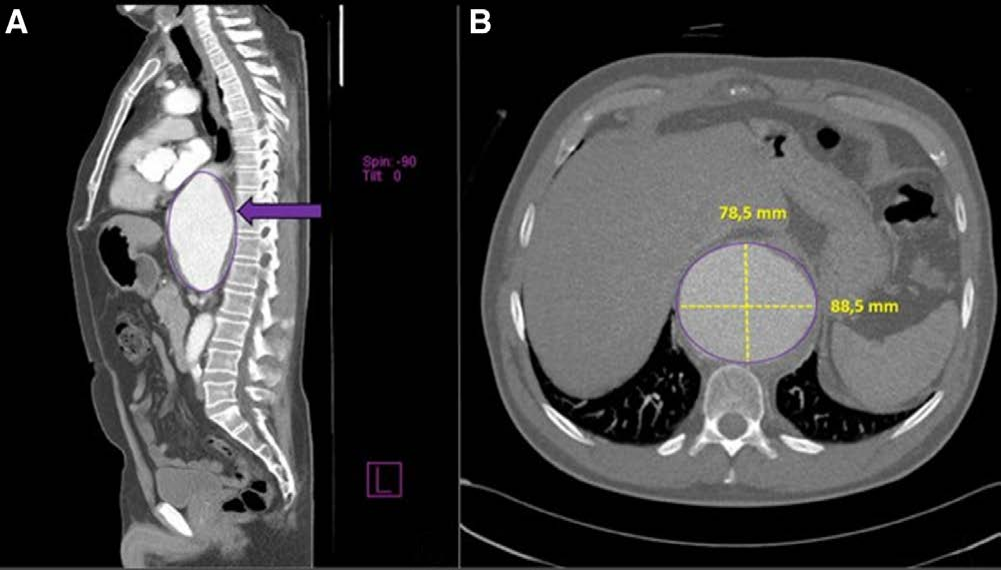
Thoracoabdominal aortic aneurysm on CT angiography imaging. Sagittal MIP reconstruction shows a fusiform thoracoabdominal aortic aneurysm (highlighted in the purple circle), with a proximal neck located in the distal segment of the descending thoracic aorta and a distal neck at the level of the origin of the SMA. A nearly circumferential mural thrombus is present (black arrow), without radiological signs of rupture (a). Axial CT image illustrating the maximum transverse dimensions of the aneurysm (purple circle) (b). CT = computed tomography, MIP = maximum intensity projection, SMA = superior mesenteric artery.

**Figure 2.**
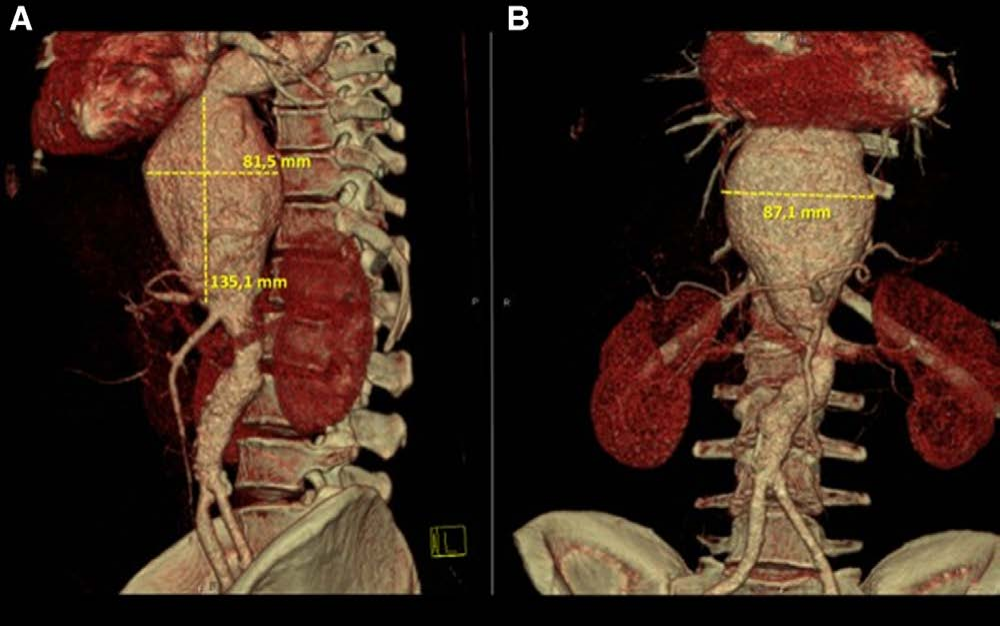
VRT of CT angiography. VRT from CT angiography showing a thoracoabdominal aortic aneurysm. Left lateral view of the thoracoabdominal aorta with evident fusiform aneurysmal dilation (A). Anterior view of the thoracoabdominal aorta demonstrating the full craniocaudal extent of the aneurysm (B). CT = computed tomography, VRT = volume-rendered 3D reconstruction.

Given the persistence of pain despite combined pharmacologic therapy, CT angiography of the aorta was performed. The examination revealed a large descending thoracoabdominal aortic aneurysm (Fig. 1), beginning just below the aortic arch and extending caudally for 14 cm, with a maximal diameter of 92 mm at the level of the diaphragm. A nearly circumferential mural thrombus measuring up to 12 mm in thickness was present, without signs of active contrast extravasation. The aneurysmal dilation caused significant compression of the posterior wall of the left atrium, inferior vena cava, distal esophagus, and cardia. Mild fusiform dilation of the subrenal abdominal aorta was also observed (Fig. 2).

The patient subsequently underwent elective surgical repair via thoraco-phreno-laparotomy. On arrival to the operating room, the patient was hypotensive (noninvasive BP 80/40 mm Hg) while receiving norepinephrine at 0.04 µg/kg/min. The aneurysm was resected and replaced with a 22-mm aorto-aortic Dacron graft. His postoperative course was uneventful, and he was discharged on postoperative day 7 in stable condition. The patient died 6 months after the event under tragic circumstances.

### 2.2. *Case report 2* – *ascending aortic dissection (Stanford type A) with atypical musculoskeletal presentation*

**Figure 3.**
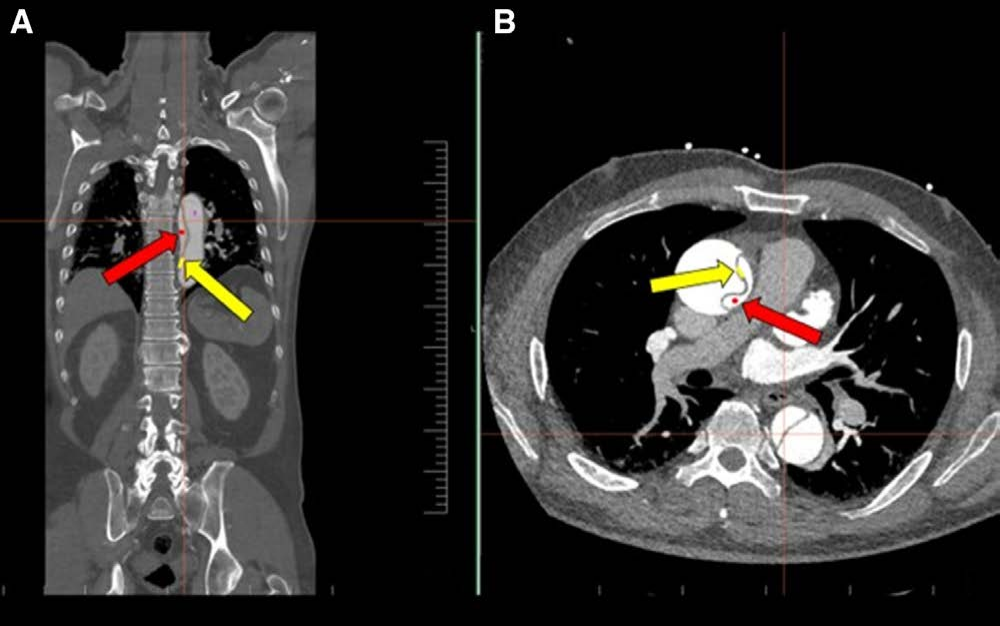
MIP reconstruction of CT angiography in the coronal (A) and transverse plane (B). Stanford type A aortic dissection is demonstrated, with a thin intimal flap (yellow arrow) visible within a fusiformly dilated ascending aorta. A slit-like, compressed true lumen (red arrow) is seen in the ascending aorta (B) and throughout the markedly dilated descending thoracic aorta (A). CT = computed tomography, MIP = maximum intensity projection.

**Figure 4.**
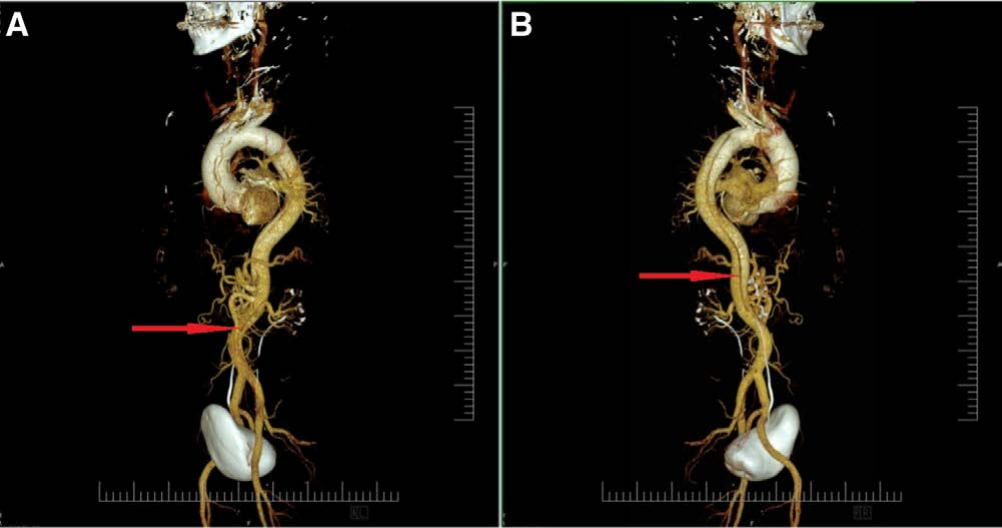
VRT of CT angiography. Stanford type A aortic dissection with an intimal flap and entry tear (yellow arrow) originating in the region of the right coronary sinus (A). The dissection extends through the aortic arch, descending thoracic aorta, and abdominal aorta, reaching the iliac arteries (blue arrow – dissection of abdominal aorta) with a reentry point in the common femoral artery (not shown). Propagation of the dissection flap into the aortic arch branches, with involvement of the left subclavian artery (B). CT = computed tomography, VRT = volume-rendered 3D reconstruction.

A 35-year-old otherwise healthy man with a history of chronic vertebrogenic pain was evaluated for gradually worsening dull pain in the cervical and thoracic spine regions, persisting for approximately 2 weeks. The pain was of moderate intensity (NRS 4–5/10), non-radiating, and unaccompanied by neurological deficits. It was intermittent, exacerbated by physical exertion, and occasionally occurred at night, disturbing sleep. Initial evaluation by a general practitioner, followed by a neurologist, led to a working diagnosis of cervical vertebrogenic syndrome. The patient was prescribed oral analgesics (paracetamol/tramadol 37.5/325 mg twice daily) and a series of diclofenac sodium/orphenadrine citrate infusions. Due to persistent symptoms and inadequate response to treatment, a repeat neurological assessment was performed. Gabapentin (300 mg every 8 hours) was added to target a potential neuropathic component. Three days later, a CT scan of the cervical and thoracic spine was performed. Although it showed no significant vertebral pathology, an incidental finding raised suspicion of aortic arch dissection. Subsequent CT angiography confirmed a Stanford Type A aortic dissection, with the intimal tear located in the right sinus of Valsalva (Fig. 3). The dissection extended into the right common carotid artery and continued to the C1 segment of the internal carotid artery. A thrombosed false lumen was observed in the left common carotid artery. Further extension involved the brachiocephalic and subclavian arteries as well as the descending aorta, reaching both femoral arteries (Fig. 4).

The patient was urgently transported via air medical service to a cardiac surgery center, where emergent surgical repair was undertaken. The patient was admitted to the operating room in an emergent setting. As he was transferred directly from the departments of radiology and neurology, no laboratory results were initially available. On arrival, he was normotensive, with noninvasive blood pressure readings of 120/70 mm Hg in the right arm and 110/65 mm Hg in the left arm, showing no significant inter-arm difference. Arterial and venous blood samples were subsequently obtained for laboratory evaluation, including complete blood count, biochemistry, coagulation profile, and blood typing. The patient was receiving a continuous infusion of isosorbide dinitrate (2 mg/hour). After induction of general anesthesia, both radial arteries were cannulated for invasive monitoring. Intraoperative TEE revealed a normally functioning aortic valve, a markedly dilated aortic root, and an intimal flap extending through the ascending and descending aorta. The procedure involved replacement of the ascending aorta with a linear interposition graft and implantation of an Evita prosthesis. The surgery was complicated by massive bleeding, necessitating repeated transfusions of blood products and coagulation factors, including recombinant activated factor VII. Due to failure to wean from cardiopulmonary bypass, veno-arterial extracorporeal membrane oxygenation (VA-ECMO) was initiated. The patient required high-dose inotropic and vasopressor support, and surgery was concluded with an open chest due to ongoing uncontrollable hemorrhage.

In the ICU, the patient received advanced critical care, including continuous analgesosedation, mechanical ventilation, continuous veno-venous hemodiafiltration (CVVHD), and ongoing ECMO support with a flow of 4.5 L/min. Despite maximal supportive therapy, his course was complicated by systemic inflammatory response syndrome, acute renal failure, and repeated reoperations for surgical bleeding. On the first postoperative day, he developed profound circulatory shock. Refractory hypotension led to ECMO failure and subsequent asystole. The patient died shortly after surgery.

## 3. Discussion

The 2 presented case reports illustrate the diagnostic challenge posed by AAS, whose manifestations are often nonspecific. Patients with dissection or aneurysm typically report chest, back, or abdominal pain, which can mimic more common musculoskeletal or visceral conditions. Up to one-third of dissections are initially misdiagnosed, most frequently as myocardial infarction, stroke, pulmonary embolism, gastrointestinal disorders, or musculoskeletal pain. Similarly, AAA, particularly when leaking or ruptured, may present with flank or groin pain that resembles renal colic.^[12]^ This wide variability underscores the need for a high index of suspicion in patients with cardiovascular risk factors and unexplained pain features.

Given these diagnostic pitfalls, current European and American guidelines emphasize structured diagnostic pathways. Risk assessment should integrate predisposing conditions (e.g., Marfan syndrome, family history, known aneurysm), pain characteristics (abrupt, tearing, or sharp), and physical findings such as pulse deficits or blood pressure differentials.^[10,11]^ In high-risk patients, immediate imaging is mandatory. Computed tomography angiography remains the preferred modality, while bedside transesophageal echocardiography (TEE) provides a reliable alternative in unstable patients. Transthoracic echocardiography (TTE) is less sensitive but may detect complications such as pericardial tamponade.^[12]^ Laboratory testing can support clinical algorithms: combining a low Aortic Dissection Detection Risk Score (ADD-RS ≤ 1) with a negative D-dimer (<500 ng/mL) safely excludes dissection in low-probability patients, though limitations remain in distal dissections, thrombosed false lumens, and intramural hematomas.^[10,11]^

Therapeutic strategies are guided by lesion type, location, and severity. Stanford type A dissections require urgent surgery, as mortality approaches 60% within 48 hours if untreated.^[10–12]^ Type B dissections are treated medically unless complicated by rupture, malperfusion, or refractory pain, where intervention is indicated.^[12]^ Aneurysm management also depends on location: ascending and arch aneurysms need open surgery, while descending thoracic and abdominal aneurysms may be treated endovascularly in suitable cases. In rupture, open repair remains standard, although the 2019 ESVS guidelines endorse EVAR in eligible patients, with trials showing outcomes equal or superior to open surgery, particularly for short-term survival.^[11,12]^

Beyond technical considerations, outcomes increasingly depend on systemic solutions. Delays in diagnosis and inappropriate therapies, such as thrombolysis for presumed myocardial infarction, highlight the importance of structured diagnostic algorithms. The ESC and ACC/AHA guidelines stress systematic risk stratification and rapid imaging, while also emphasizing the role of Multidisciplinary Aortic Teams, which integrate surgeons, cardiologists, radiologists, anesthesiologists, and intensivists.^[13,14]^ Evidence demonstrates that care in high-volume centers with such teams improves diagnostic accuracy, accelerates decision-making, and reduces perioperative mortality.^[12,15,16]^

In our series, outcomes differed markedly: the patient with the abdominal aortic aneurysm achieved full postoperative recovery and was discharged in stable condition, whereas the patient with Stanford type A dissection developed severe postoperative complications including systemic inflammatory response syndrome, acute renal failure, and refractory circulatory shock, resulting in death within 24 hours after surgery. Although diagnostic delay is a well-recognized risk in AAS, our cases do not allow a direct causal link to be established. Current evidence shows that surgical outcomes are best in patients treated within the first hours or, in some cases, after a later stabilization period. These reports therefore underscore the importance of maintaining a high index of suspicion and applying structured diagnostic pathways when evaluating patients with nonspecific chest, back, or abdominal pain.

## 4. Limitations

This case series is limited by its descriptive nature and the absence of long-term follow-up data. Causal relationships between delayed diagnosis and outcomes cannot be established. Furthermore, as both cases were analyzed retrospectively, no additional confirmatory investigations were performed beyond the available clinical documentation.

## 5. Conclusion

AAS, including dissection and aneurysm, often present with deceptively nonspecific symptoms that mimic more common musculoskeletal or visceral conditions. The first case illustrates a clinically silent abdominal aneurysm misinterpreted as vertebrogenic back pain, successfully treated once vascular etiology was recognized. The second case highlights the extreme diagnostic challenge of ascending aortic dissection in a young patient presenting with atypical symptoms; despite surgery, the outcome was unfavorable. Together, these cases underscore the need to maintain a high index of suspicion for life-threatening vascular disease even in patients without classic risk profiles.

In our series, outcomes differed: the patient with Stanford type A dissection did not survive, whereas the patient with abdominal aneurysm was successfully treated. Although diagnostic delay is a well-recognized risk factor, these reports do not establish a direct causal link. Current evidence suggests that the best surgical outcomes occur in patients treated within the first hours or, in some cases, after a later stabilization period.

## 6. Lessons learned

- Nonspecific pain may conceal life-threatening aortic syndromes and should be considered in the differential diagnosis of chest, back, or abdominal pain.
- Structured diagnostic pathways (e.g., ADD-RS, D-dimer, prompt imaging) help reduce misdiagnosis and delays.
- Optimal outcomes require coordinated multidisciplinary aortic team care and early referral to specialized centers.

## Author contributions

**Conceptualization:** Ladislav Kočan, Alena Šujanová, Janka Vašková.

**Data curation:** Ladislav Kočan, Dušan Rybár, Adrián Kolesár, Štefan Lukačin, Alena Šujanová, Andrea Galovičová, Jozef Židzik.

**Formal analysis:** Ladislav Kočan, Dušan Rybár, Adrián Kolesár, Štefan Lukačin, Alena Šujanová, Andrea Galovičová, Jozef Židzik, Janka Vašková.

**Investigation:** Dušan Rybár, Štefan Lukačin, Alena Šujanová.

**Methodology:** Ladislav Kočan, Adrián Kolesár, Štefan Lukačin.

**Supervision:** Ladislav Kočan, Janka Vašková.

**Validation:** Ladislav Kočan, Dušan Rybár, Adrián Kolesár.

**Visualization:** Štefan Lukačin, Andrea Galovičová.

**Writing – original draft:** Ladislav Kočan, Janka Vašková.

**Writing – review & editing:** Ladislav Kočan, Dušan Rybár, Adrián Kolesár, Štefan Lukačin, Alena Šujanová, Andrea Galovičová, Jozef Židzik, Janka Vašková.
